# Typhoid Fever and London Water

**Published:** 1897-10-16

**Authors:** 


### The Hospital

A Journal of

The Medical Sciences and Hospital Administration.

Vol. XXIII.—No. 577.] [Oct. 16, 1897.

### Typhoid Fever and London Water.

The terrible outbreak of typhoid fever which has devastated the fair town of Maidstone ought to impress upon the minds, even of the most careless, the importance of securing a pure water supply for the use of every town.

The fact that if water is polluted with typhoid excreta it becomes itself infectious, and capable of distributing the disease among all who drink it, is so well known that we are apt, perhaps not unnaturally, to take for granted that those in power—"the authorities," whoever they may happen to be—will exercise the strictest supervision over all that appertains to its purity. Vain hope! What has happened at Maidstone is but an example of what is going on all around, and many a town is drinking water just as unsafe, and only escapes fever by mere accident. We may at once say that people living in London have no reason to fear any outbreak of typhoid comparable at all to that at Maidstone. The conditions of the water supply are absolutely different, and the intensity of infection which is possible enough in a well, or in such a case as the well-known Caterham outbreak, is absolutely inconceivable in regard to great rivers like the Thames or Lea. For all that, however, we always have more or less typhoid among us, and people may fairly ask what security they have that the means adopted to protect them from it are sufficient, and are properly maintained.

That this fever is constantly present in the area from which the London water supply is drawn is certain. On the other hand, it is equally certain that the typhoid rate in London is low. With this many are content, but all cautious people would like to know whether the low typhoid rate in London is due to good luck, as has been the case with other towns, which, after years of freedom, have been attacked by great epidemics, or is due to good management on which they can safely rely for protection from so insidious a foe. After very careful consideration of this question our answer is that luck has had a great deal to do with the comparative immunity which we have enjoyed. The means adopted by the companies are no doubt efficient so far as they go, but in no case is there any guarantee that they are sufficient or are kept in proper working order.

This is really the crux of the whole matter. No doubt, on the average, the London water is of a high degree of purity, but it should be remembered that it is but a small advantage to be supplied with pure water "as a general thing" if now and again it comes to us charged with infection. The purity of a water supply is to be measured by the worst sample, not by the best. In times of flood, when the Thames becomes a turbid river charged with the filth of all the districts which it drains, including many large towns, every purifying agency except filtration fails entirely, and nothing but the efficiency of the filtering process stands between London and water-borne disease.

To emphasise this still further we would point out that neither the water companies who are responsible for supplying a pure and potable water, the Local Government Board who are responsible for its analysis, nor the County Council who represent those who drink it, have any control over the river from which it is drawn. This lies in the hands of the Thames Conservancy Board, a body principally concerned with the navigation of the river.

The London companies have to take the water as it is served to them—to take it or leave it. Just the same thing happens to the consumer. He also has to take it or leave it, with the additional disadvantage of having to pay for it in either case; and, strange to say, even the County Council, acting for their electors, also can but take it or leave it, for even they have no control over the filtering process, and have not even the right to send a man to see whether the filters are clean or dirty.

What guarantee have we, then, of the condition of the London water? A monthly report! And that by an examiner who is only allowed on the companies' premises as a matter of courtesy!! But even that does not fully express the laxity of our arrangements in regard to water. Some of the companies run all their filtered water into a general receptacle from which the examiner takes his monthly sample. What is badly filtered is mixed with what is well filtered, and thus its default is covered up. Take one of the very best of the Thames companies, one which has been able on an average to remove 98·87 per cent. of all the microbes. Sometimes it has removed even 99·94 per cent., and perhaps more, but at other times only 92·67 per cent., allowing upwards of 7 per cent. of the microbes it contained to pass through. There is nothing, however, to show that this latter condition was due to a general deterioration all over the filter, and—for all we know to the contrary—it may but indicate that 7 per cent. of the water pouring into the mains was entirely unfiltered.

That this is the real explanation is rendered almost certain by the results of independent inquiries made on behalf of the London County Council, whose chemist, by submitting the water from the mains to careful filtration, fished out of it entozoic worms, vegetable fibres, and numerous infusoria many times larger than the largest bacteria. Dr. Klein also reported to the same effect, saying that he was able to find in the water "briskly-moving animalculi," which "must have been derived from the water and have passed through the filters." Mr. Shirley Murphy again has shown that an abnormal distribution of typhoid fever in the part of London supplied with Thames water has followed an abnormally high flood in the Thames valley; and, putting all the evidence together, the presumption is very strong that the filtration which is our sole protection from Thames typhoid is sometimes only very imperfectly performed. Certainly no efficient and continuous inspection is made as to the action of each filter, and no official inspector is armed with authority to stop the distribution of water from a filter which is found to be defective. There is no one to turn the tap and stop the supply from a bad filter. This, we think, is a serious blot upon our sanitary machinery, and the gravity of the matter seems in no way lessened by the fortunate accident that so far no very obvious evil has resulted.

